# Forensic genetic analysis of population of Madhya Pradesh with PowerPlex Fusion 6C^™^ Multiplex System

**DOI:** 10.1007/s00414-019-02017-0

**Published:** 2019-02-14

**Authors:** Shivani Dixit, Pankaj Shrivastava, R. K. Kumawat, Kamlesh Kaitholia, Hirak Ranjan Dash, Harsh Sharma, Gyaneshwer Choubey

**Affiliations:** 1 DNA Fingerprinting Unit, State Forensic Science Laboratory, Department of Home (Police), Government of MP, Sagar, 470001 India; 2 DNA Division, State Forensic Science Laboratory, Jaipur, Rajasthan 302016 India; 3 Cytogenetics Laboratory, Department of Zoology, Banaras Hindu University, Varanasi, 221005 India

**Keywords:** Forensic, DNA fingerprinting, STR, Madhya Pradesh, India, PowerPlex fusion 6C

## Abstract

Performance of PowerPlex Fusion 6C kit (PP F6C) was assessed in 374 unrelated individuals belonging to Madhya Pradesh, an Indian state. The study evaluated the forensic parameters for the loci included in PP F6C Multiplex System. The combined discrimination power (CPD) and combined exclusion power (CPE) were 1 and 0.999999995, respectively, for all 23 autosomal STR loci. SE33 showed the greatest power of discrimination (0.990) in the studied population, whereas TPOX showed the lowest (0.843). The availability of three Y-STR loci in the Multiplex System is suitable for assessing male contribution and amelogenin deletion in a single Multiplex PCR simultaneously. The study also presents the first global report on polymorphism in the Indian population on SE 33 autosomal STR loci and PP Fusion 6C Multiplex System. The results revealed that the studied STR Multiplex System is highly polymorphic and suitable for forensic purposes.

**Electronic supplementary material:**

The online version of this article (10.1007/s00414-019-02017-0) contains supplementary material, which is available to authorized users.

## Description

Present capillary electrophoresis and PCR-based STR analysis using expanded Combined DNA Index System (CODIS) 20 markers are state of art in forensic DNA typing [1]. For performing the identification conclusively and precisely with a significant probability value, an increase in the number of loci is recommended, as the discrimination power of any Multiplex System is based on a combination of all the independent loci tested. Madhya Pradesh is the second largest state in India by area. The population of Madhya Pradesh is 72,597,565 comprising of 37,612,920 males and 34,984,645 females, contributing 6% to India’s total population as per the 2011 census. A few studies have been performed to characterize the population of Madhya Pradesh on autosomal STRs (described in Table S2) but these studies are based on 15 STR markers either using Identifiler/Identifiler Plus (Applied Biosystem, USA) or PowerPlex 16/16HS (Promega Corporation, Madison, USA).

Based on six dyes, PP F6C System (Promega Corporation, Madison, USA) is a 27-locus multiplex that ensures simultaneous detection of 20 autosomal loci in the expanded CODIS (CSF1PO, FGA, TH01, TPOX, vWA, D1S1656, D2S1338, D2S441, D3S1358, D5S818, D7S820, D8S1179, D10S1248, D12S391, D13S317, D16S539, D18S51, D19S433, D21S11, and D22S1045), three more loci for better discrimination (Penta D, Penta E and SE33), Amelogenin for sex determination and DYS391 and two rapidly mutating Y-STR loci, DYS570 and DYS576 for further confirmation of gender. A multi-laboratory evaluation of the PP F6C System has already been published [2] following SWGDAM guidelines [3]. This study is the first global attempt to work out the efficacy of PP F6C System and the first ever population data statement about SE 33 marker in Indian population for forensic purposes. Samples (*n* = 374) of unrelated individuals were taken from routine casework analysis performed between January 2018 and October 2018 by the authors who work at the DNA Fingerprinting Unit, State Forensic Science Laboratory, Sagar, Madhya Pradesh, India. The samples included in this study have not been typed by any other Multiplex System and this is the first study from India detailing the forensic efficacy of a 27-marker multi-utility STR Multiplex System. Following the Code of Ethics of the World Medical Association (i.e., Declaration of Helsinki) for experiments involving humans, peripheral blood samples of these unrelated individuals were collected. All individuals provided prior written informed consent. No minor was involved in the study.

After DNA extraction and PCR, analysis was performed on 3500XL genetic analyzer, and statistical evaluation was done as described before [4]. The peak height threshold was 50 RFU for heterozygous and 200 RFU for homozygous alleles. Internal laboratory control standards and kit controls were used at all steps of analysis. The authors have passed the proficiency test of the GITAD, Spain (http://gitad.ugr.es/principal.htm). This article follows the population data publication guidelines formulated by the journal.

The allele frequencies and results of the forensic efficiency parameters for the 23 autosomal STR loci under study are given in Table S1. The frequency values observed in this study were compared with the previously published data (Table S2) on 17 autosomal STR markers from India and with recently published data on 22 autosomal STR markers [5].

**Fig. 1:**
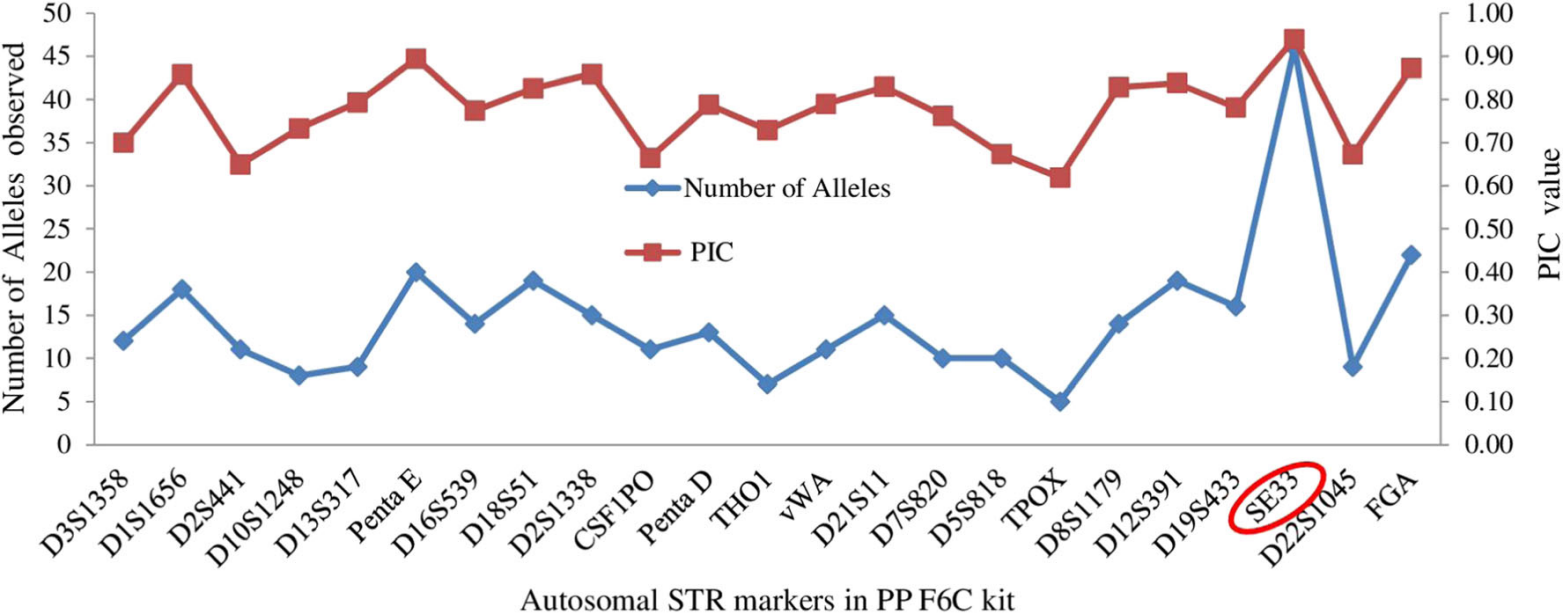
Number of alleles observed in PP F6C Multiplex System and PIC in population of Madhya Pradesh

A total of 334 alleles were observed in the population of Madhya Pradesh, with SE 33 possessing the highest (46) number of alleles and TPOX with lowest (05) number of alleles (Table S1 and Fig. 1). Of the 374 samples tested, one sample showed amelogenin deletion, with X-profile on amelogenin marker and deletion on DYS576 and DYS570 marker with amplification on DYS391 marker (Fig. S1).

**Fig. 2:**
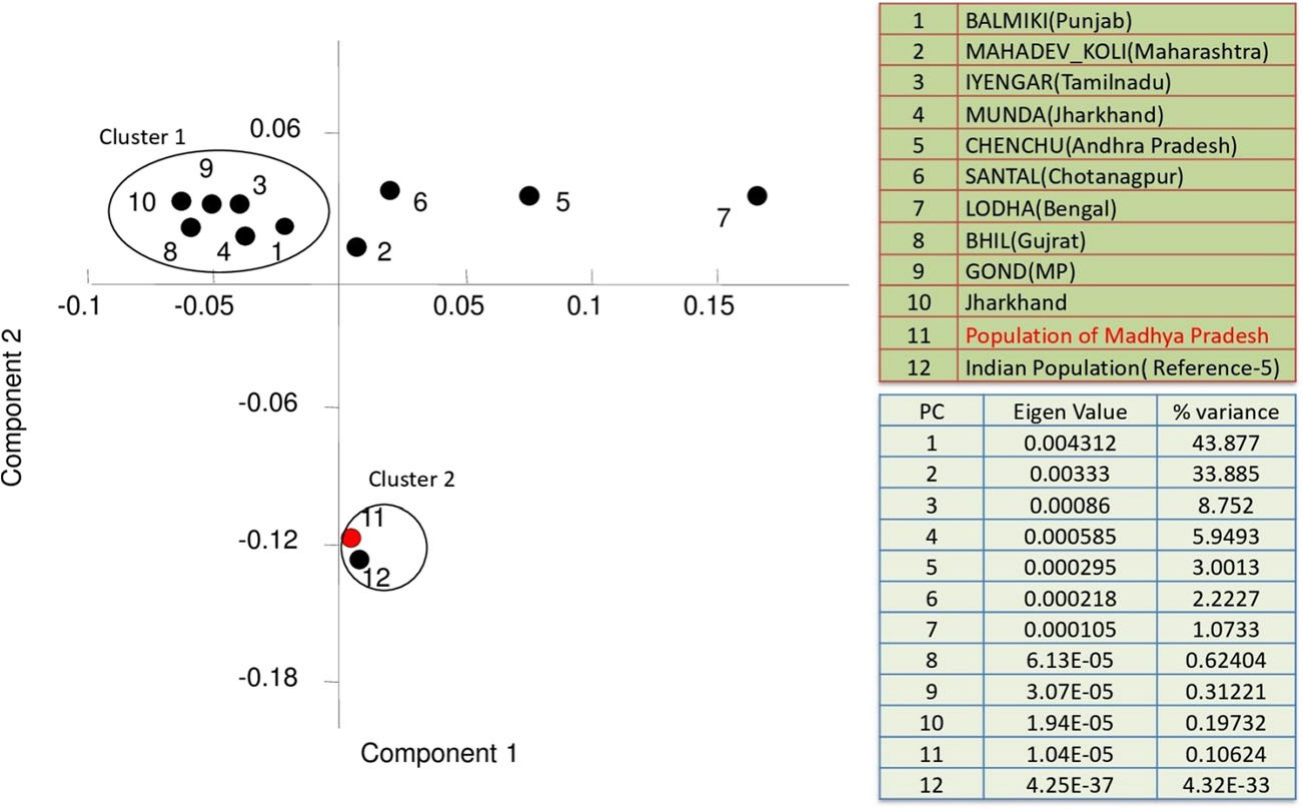
PCA plot of Madhya Pradesh population with the compared populations based on available 15 autosomal STR data

**Fig. 3:**
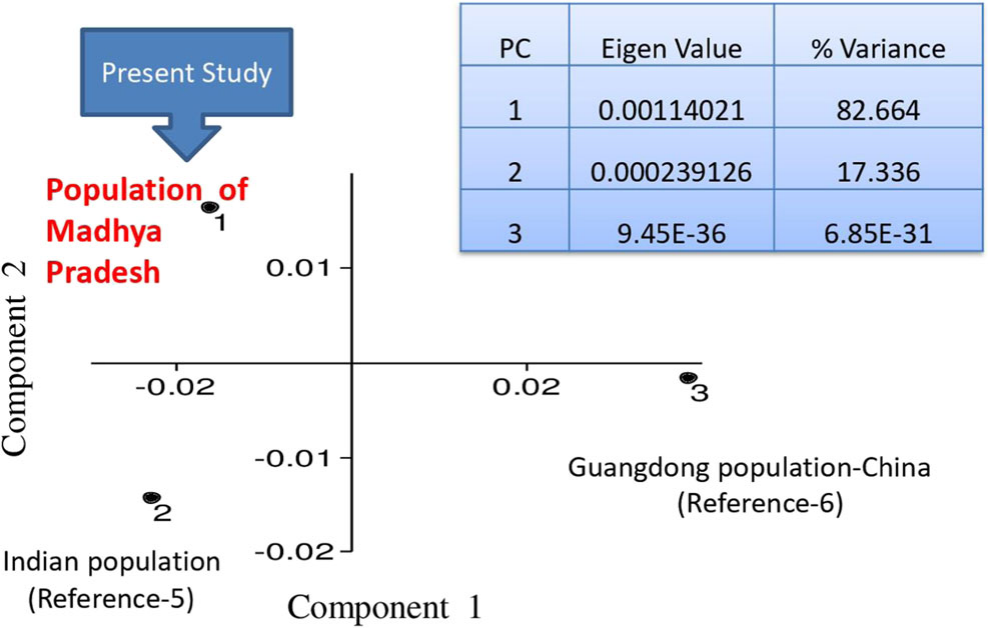
PCA plot showing comparison of Madhya Pradesh Population with the published populations based on 22 common autosomal STR data

The studied population followed the Hardy-Weinberg equilibrium for all loci except for D21S11, D7S820, TPOX, D1S1656, D2S441, D10S1248, and FGA after the Bonferroni correction. The observed and expected heterozygosity in the studied Multiplex System ranged from 0.577 to 0.877 and 0.661 to 0.939, respectively. The most polymorphic and discriminatory STR loci in the studied population were SE 33 with values of 0.94 and 0.990, respectively. All the loci included in the studied Multiplex System were found to be polymorphic (PIC value = < 0.62 to 0.94). The power of exclusion (PE) ranged from 0.331 (D22S1045) to 0.770 (SE 33) with a value of greater than 0.999999995 for the combined power of exclusion (CPE). The power of discrimination (PD) ranged from 0.843 (TPOX) to 0.990 (SE 33) with a value of one for the combined power of discrimination (CPD). The combined probability of the match (CP_m_) and combined paternity index (CPI) for all 23 autosomal STR loci were found to be 4.4 × 10^−29^ and 1.47 × 10^8^, respectively. Therefore, the 23 autosomal STR loci exhibited high efficacy and informativeness for forensic investigation and individualization purposes. The data obtained in this study were compared with published Indian population data (Table S2) related to common 15 autosomal STR loci. Pairwise Fst matrix between the studied population (marked as Madhya Pradesh population) and reference populations at the common 15 loci have been shown in Table S2. NJ tree and PCA plot are given in Fig. S2 and Fig. 2, respectively. The distribution pattern of populations demonstrated that the genetic affinity has existed within ethnic origins and geographically close populations. The studied population was also compared with previously published data of Indian population [5] and Guangdong population of China [6] at common 22 loci and NJ tree was constructed using Poptree2 Software. The studied population (marked as Madhya Pradesh population) made a pool with previously published Indian population reflecting the same origin and Guangdong population of China was found an outlier (Fig. 3). PCA plot drawn with the same populations showed that all the compared populations are genetically distant. Indian population [5] and the studied population have the same origin but during evolution, they became genetically different which is reflected in PCA plot. Thus, the NJ tree and PCA analyses, both were consistent with our observations in the studied population. For the Y-linked loci DYS391, DYS570, and DYS576, estimated heterozygosity was observed as 0.32, 0.79, and 0.81 respectively (Fig. S3). Mean genetic diversity and mean genetic distance between individuals based on the cumulative values observed by the analysis of three Y-STRs included in PP F6C were found to be 0.973 and 5.838, respectively.

The study will add to the data bank of various studies conducted on the Indian population. With respect to the distribution of alleles at each STR locus, the loci were found to be substantially polymorphic in this population indicating good informativeness of all studied autosomal STR markers. In addition, the Multiplex System is found suitable in deciphering the amelogenin deletion to avoid misinterpretation and/or to assess presence of male DNA (if the DNA is male mixed). The interested researchers can request the data from the authors.

## Electronic supplementary material

ESM 1 (XLSX 21.5 kb)

ESM 2 (XLSX 11.0 kb)

ESM 3 (XLSX 13.0 kb)

ESM 4 (DOCX 327 kb)
