# Supplementary material for: Forensic genetic analysis of population of Madhya Pradesh with PowerPlex Fusion 6C™ Multiplex System
Source: Int J Legal Med. 2019 Feb 14;133(3):803–5. doi: 10.1007/s00414-019-02017-0 (PMC6469663; doi:10.1007/s00414-019-02017-0)

Figure S1: Autosomal STR DNA Profile of one sample showing Amelogenin deletion in Madhya Pradesh Population.


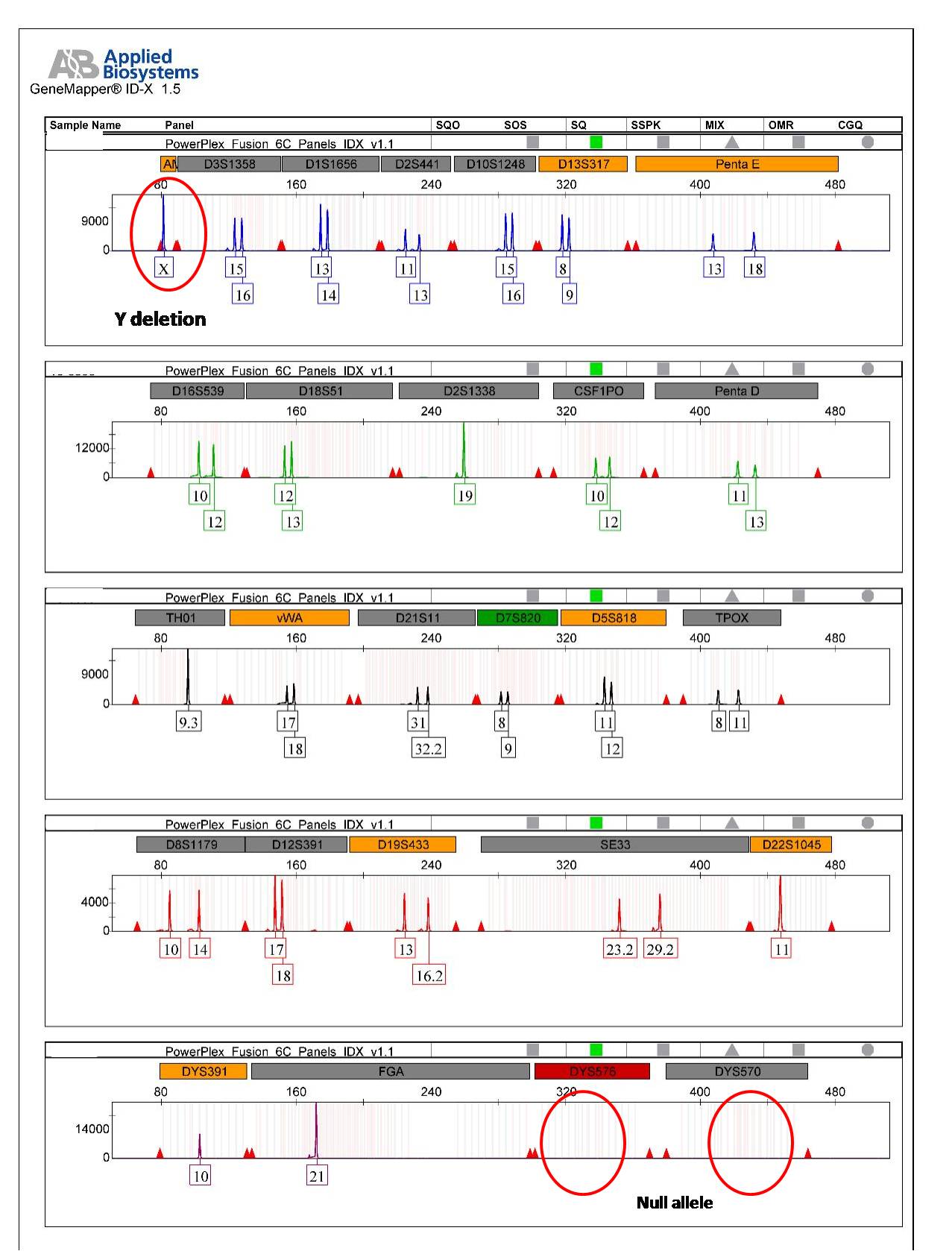


Figure S2. NJ tree comparison of Madhya Pradesh with other reported populations based on autosomal STR data.


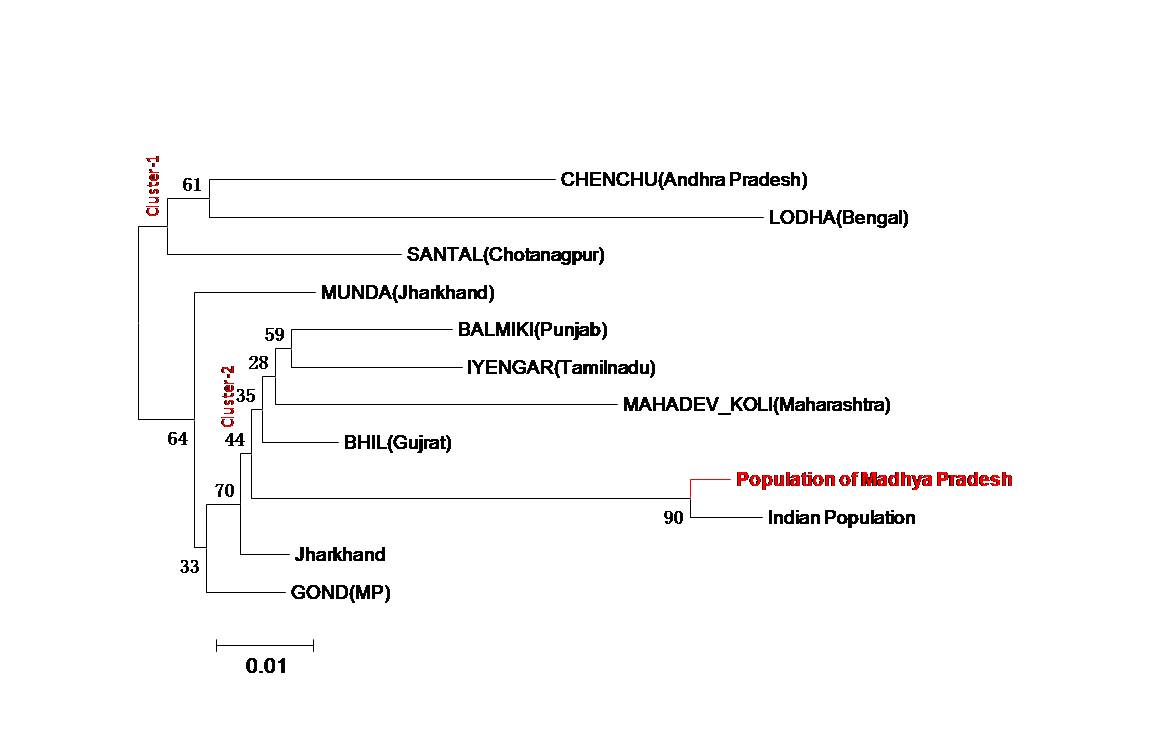


Figure S3. Genetic Diversity of three Y-STR Loci included in PP F6C Multiplex System in Population of Madhya Pradesh.


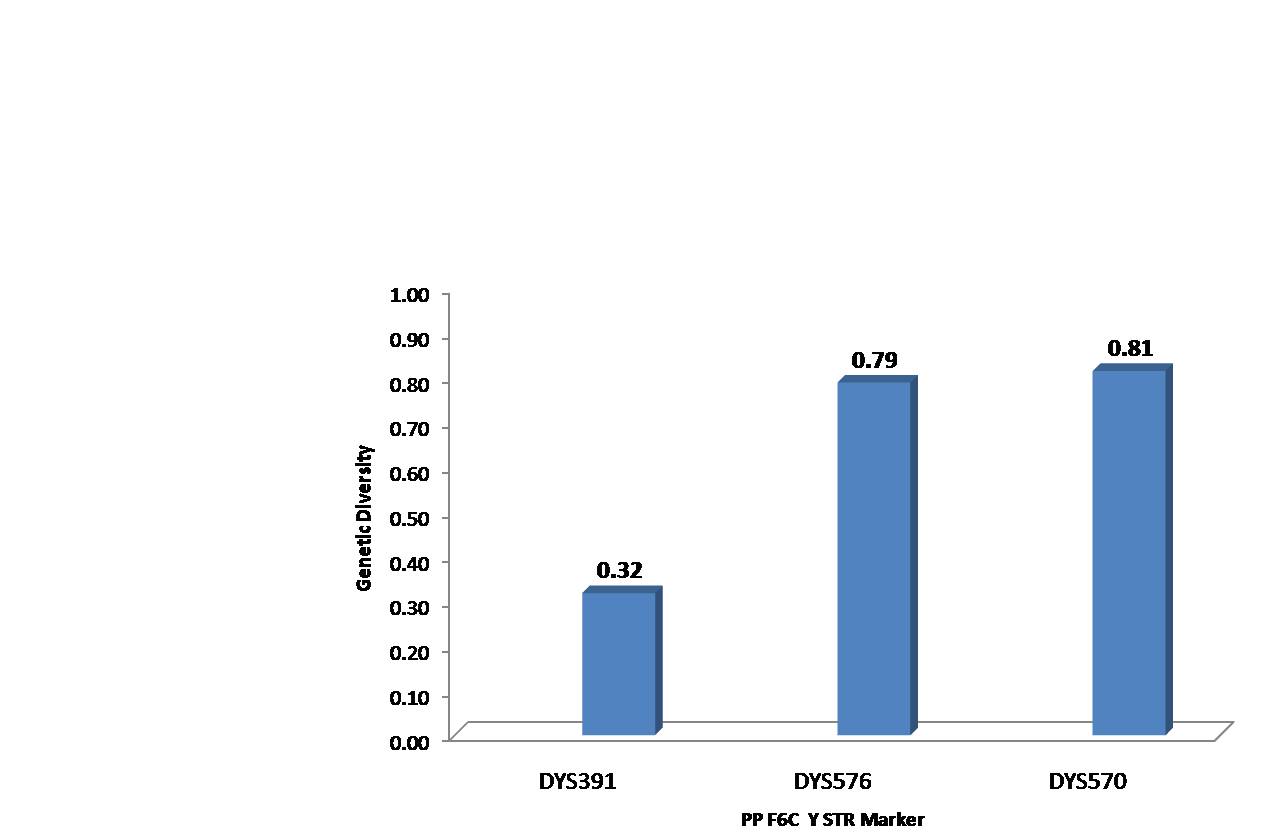

Supplement: Supplementary file 4 — (DOCX 327 kb) [file 414_2019_2017_MOESM4_ESM.docx]
